# Characterization of a new IN-I-PpoI fusion protein and a homology-arm containing transgene cassette that improve transgene expression persistence and 28S rRNA gene-targeted insertion of lentiviral vectors

**DOI:** 10.1371/journal.pone.0280894

**Published:** 2023-01-20

**Authors:** Alisa Nousiainen, Diana Schenkwein, Seppo Ylä-Herttuala

**Affiliations:** 1 A. I. Virtanen Institute, University of Eastern Finland, Kuopio, Finland; 2 Heart Center and Gene Therapy Unit, Kuopio University Hospital, Kuopio, Finland; Hirosaki University Graduate School of Medicine, JAPAN

## Abstract

Targeting transgene integration into a safe genomic locus would be very important for gene therapy. We have generated lentivirus vectors containing the ribosomal RNA-recognising I-PpoI endonuclease fused to viral integrase, and transgene cassettes with target site homology arms to enhance insertion targeting. These new vectors were characterised with respect to the persistence of transgene expression, insertion targeting efficiency and chromosomal integrity of the transduced cells. The aim was to find an optimally safe and effective vector for human gene therapy. Fusion protein vectors with high endonuclease activity were the most effective in the accurate targeting of transgene insertion. The homology construct increased the insertion targeting efficiency to 28% in MRC-5 cells. However, karyotyping analysis showed that the high endonuclease activity induced the formation of derivative chromosomes in as many as 24% of the analysed primary T lymphocytes. The persistence of transgene expression was excellent in homology arm-containing fusion protein vectors with reduced endonuclease activity, and these fusion proteins did not cause any detectable chromosomal rearrangements attributable to the endonuclease activity. We thus conclude that instead of the fusion protein vectors that carry a highly active endonuclease, our vectors with the ability to tether the lentivirus preintegration complex to benign loci in the genome without high ribosomal DNA cleavage activity are better suited for lentivirus-based gene therapy applications.

## Introduction

Curative gene therapy for monogenic diseases is an important goal for molecular medicine. Lentivirus vectors (LVs) are in many ways ideal for this purpose, as they can carry large transgene constructs to a wide variety of cell types and generate long-lasting transgene expression [1, 2]. Most currently used LVs are based on human immunodeficiency virus type 1 (HIV-1) and integrate preferentially into transcriptionally active regions of protein-encoding genes [3, 4]. However, the ability to integrate transgenes poses an inherent risk of genotoxic effects; in LVs, the risks are considered to be mainly related to insertional inactivation and gene truncation [5].

Our approach for targeted LV integration is the use of the I-PpoI homing endonuclease fused to HIV-1 viral integrase (IN) protein [6–9]. Like other nuclease-based targeting approaches such as TAL-effector nucleases, zinc-finger nucleases, and the CRISPR/Cas9-system (the clustered regularly interspaced short palindromic repeats/CRISPR-associated nuclease 9), the catalytically active IN-I-PpoI-fusion proteins target transgene insertion by creating targeted double-strand breaks (DSBs). An exogenous DNA template can be inserted into the break site by the target cell’s endogenous DNA repair machinery. Depending on the sequence and delivery method of the template, the insertion can be mediated by non-homologous end-joining (NHEJ) or homologous recombination (HR) [10].

I-PpoI has an endogenous recognition sequence in the 28S ribosomal RNA (rRNA) gene, which is in many ways a desirable integration target: its function is unlikely to become abolished as the rRNA genes are present in hundreds of copies per cell, and the ribosomal DNA (rDNA) is located far from potentially oncogenic protein-encoding genes [11]. However, the rDNA repeat sequence also poses some challenges as an insertion target. We have seen that lentiviral delivery of a highly active I-PpoI endonuclease reduces the number of viable cells in culture due to excess rRNA gene cleavage [6, 8]. In addition, the assessment of targeted insertion efficiency with sequencing methods based on PCR becomes challenging when the target site is present in hundreds of copies per cell and insertion targeting is very efficient. In a highly targeted locus, a “saturation effect” hampers the differentiation of similar reads from PCR-borne replicates, leading to an underestimation of the targeting efficiency.

In this study, we describe new vectors designed to create ideal levels of target DNA cleavage, insertion targeting and transgene expression by the IN-modified LVs. We designed a new LV transgene construct, which was appended with rDNA-compatible homology arms (HA) to enhance targeted insertion. This construct was used in combination with both a new IN-I-PpoI fusion protein and our previously characterised LVs, in which I-PpoI has the endonuclease-inactivating mutation N119A or an activity-reducing H78A-mutation [6–8]. IN-I-PpoI_N119A_ improves the safety of lentiviral integration by reducing transgene insertion into unique protein-encoding genes, and IN-I-PpoI_H78A_ efficiently directs vector insertion into rDNA through DSB repair [6, 7]. Since excess DSB formation can be harmful for cells, the new IN-I-PpoI_R61A+H78A_ fusion protein LV (RH LV) was created to study whether efficient targeted integration into the I-PpoI cleavage site can also be achieved when the endonuclease activity is further reduced with an added R61A point mutation [12]. In this study, we used droplet digital PCR (ddPCR) to quantify HR events, which have been difficult to analyse even with integration site sequencing methods. Last, to get an unbiased genome-wide view of the chromosomal effects of the IN-modified LVs, we karyotyped cells transduced with different IN-modified LVs.

The main goals of the study were to characterise targeted insertion events by the new IN-I-PpoI fusion protein, the effects of the new HA construct on the functionality of the fusion protein LVs, and to assess possible structural changes caused by these LVs at the chromosomal level. We found that the addition of the homology arms in the transgene construct improved the persistence of transgene expression when combined with fusion protein LVs with reduced endonuclease activity, and that these fusion proteins did not cause detectable chromosomal rearrangements attributable to the endonuclease activity. Combining the HA construct with the fusion protein LV with a high endonuclease activity led to an increased efficiency of the insertion targeting. However, transgene expression persistence with these vectors was low, and the high endonuclease activity also led to major structural changes in the chromosomes of the transduced primary T lymphocytes. We thus conclude that IN-I-PpoI fusion proteins with low endonuclease activity are superior to the highly active endonuclease proteins in obtaining safe gene transfer and sustained transgene expression.

## Materials and methods

### Third-generation LV constructs, fusion proteins, and LV production

The LVs used in this study were third-generation LVs pseudotyped with the G protein of vesicular stomatitis virus (VSV-G). The packaging plasmids differed depending on the desired integrase modifications of the LVs; these plasmids are listed in Table 1. The packaging constructs used to produce INwt, D+H and D+N LVs were as previously described [6, 8]. For the RH LVs, a new construct was produced via synthesis of an 886 bp DNA fragment which corresponded to the sequence between the AflII and BspEI cut sites in the pMDLg-pRRE-IN-I-PpoI_H78A_ plasmid with the codon for I-PpoI aa 61 changed from CGC to GCT (R>A). This fragment was synthesized at Genewiz and then cloned to the pMDLg-pRRE-IN-I-PpoI_H78A_ plasmid to produce pMDLg/pRRE-IN-I-PpoI_R61A+H78A_, which was used in the LV production.

**Table 1:** The types of LVs, their integrase composition and packaging plasmids used in this study.

Abbreviation	Integrase composition	Packaging plasmids
**INwt**	Wild-type integrase	pMDLg/pRRE
**D+H**	IN_D64V_ + IN-I-PpoI_H78A_	pMDLg/pRRE-IN_D64V_ + pMDLg-pRRE-IN-I-PpoI_H78A_
**D+N**	IN_D64V_ + IN-I-PpoI_N119A_	pMDLg/pRRE-IN_D64V_ + pMDLg/pRRE-IN-I-PpoI_N119A_
**RH**	IN-I-PpoI_R61A+H78A_	pMDLg/pRRE-IN-I-PpoI_R61A+H78A_
**D64V**	IN_D64V_	pMDLg/pRRE-IN_D64V_

The pLV-EGFP and pLV-ZeoR have been previously described [7]. For the HA constructs, two homologous arms of 500 bp each of the genomic sequence flanking the cut site of I-PpoI in the 28S rRNA gene were added to the pLV-EGFP plasmid upstream of the hPGK promoter (upstream homology arm) and downstream of the WPRE sequence (downstream homology arm). The pLV-28S HR-EGFP-plasmid was done at Genewiz (Leipzig, Germany) by synthesis and cloning of the plasmid fragment from the beginning of the upstream HA to the end of the downstream HA. For Zeocin-selection experiments, the *sh Ble*-transgene from the pLV-ZeoR-plasmid was cloned to replace the transgene in the pLV-28S HR-EGFP to produce the pLV-28S HR-ZeoR-plasmid.

The LV production and titration was done at the Biocenter Kuopio National Virus Vector Laboratory at the A.I. Virtanen Institute for Molecular Sciences. LVs were produced following the standard calcium phosphate transfection method and concentrated by ultracentrifugation [13]. Particle titration was done using the Alliance HIV-1 p24 Antigen ELISA Kit (NEK050, PerkinElmer, Waltham, Massachusetts), and functional titration was done by flow cytometry analysis of transduced HeLa cells on day 3 post-transduction with FACSCalibur (BD Biosciences, San Jose, California).

### Fusion protein cleavage activity assessment *in vitro*

The functionality of the IN-fusion proteins used in this study was assessed with a digestion of a control plasmid using crude extracts of LV preparations as previously described [8]. The control plasmid was prepared by cloning a 367 bp PCR product of the human genomic DNA sequence flanking the I-PpoI cut site in Chr21 (5’ primer: GACTTAGAACTGGTGCGGAC, 3’ primer: CACTTATTCTACACCTCTCATG) to the pJet1.2/Blunt vector plasmid using the CloneJET PCR Cloning kit (ref. K1232, Thermo Fisher Scientific, Vilnius, Lithuania). A 30 μl aliquot of each tested LV was mixed 1:1 with lysis buffer (0.25% Igepal CA-630 (ref. 56741-50ML-F, Fluka, Buchs, Switzerland) and Complete protease inhibitor cocktail (ref. 11836145001, Roche, Mannheim, Germany) in DPBS (ref. 14190–094, Gibco, Bleiswijk, the Netherlands). 500ng of plasmid DNA was digested using 10 μl of the LV extract in 1x I-PpoI-buffer and 1x BSA and an incubation at 37°C for 90 min, with a control reaction using the I-PpoI enzyme (ref. R703A, Promega, Madison, Wisconsin). The digestion buffer was changed with the NucleoSpin Gel and PCR Clean-up kit (ref. 740609–250, Macherey-Nagel, Düren, Germany), and the samples were digested with Bsu1I (ref. FD0143, Thermo Fisher Scientific, Vilnius, Lithuania) and analysed on an agarose gel.

### Cells and culture conditions

The MRC-5 human lung fibroblast cell line (CCL-171^™^, ATCC^®^, Manassas, Virginia) was used in testing the transgene expression and targeted insertion of EGFP-containing LVs. The cells were cultured in Dulbecco’s modified Eagle’s medium (D6429, Sigma, Darmstadt, Germany) supplemented with 10% foetal bovine serum (F7524, Sigma, Darmstadt, Germany), 1% MEM non-essential amino acids (M7145, Sigma, Darmstadt, Germany), 1% sodium pyruvate (ref. 11360–039, Gibco, Bleiswijk, the Netherlands), and 1% penicillin-streptomycin (P0781, Sigma, Darmstadt, Germany).

Human T lymphocytes, extracted from leukoreduction system (LRS) chambers purchased from Finnish Red Cross Blood Service, were used to test transgene expression, targeted insertion, and chromosomal effects of LVs carrying the *Sh Ble*-transgene. From the LRS chambers, peripheral blood mononuclear cells (PBMCs) were separated using Leucosep Centrifuge tubes (ref. 227288, Greiner Bio-one, Frickenhausen, Germany), and T cells were isolated from the PBMCs using the Pan T Cell Isolation kit (ref. 130-096-535, Miltenyi-Biotech, Bergisch Gladbach, Germany). T cells were activated using Dynabeads (human T-activator CD3/CD28, ref. 11132D, Gibco, Vilnius, Lithuania). The T cells were cultured at a concentration of 1x10^6^ cells/ml in X-VIVO 15 medium (BE02-060F, Lonza, Verviers, Begium) supplemented with 5% human AB serum (S4190, Biowest, Nuaillé, France) and 20 U/ml human recombinant IL-2 (Cyt-209-b, Prospec, Rehovot, Israel).

### Flow cytometry analysis of EGFP-transgene expression

MRC-5 cells were transduced with EGFP-carrying LVs on six well plates at a concentration of 7.5K vector particles (VP)/cell based on their p24 titre, with three replicate wells of 2x10^5^ cells for each LV. The experiment was repeated three times. The cells from each well were sampled for flow cytometry analysis at days 2, 6, 9, and 13 post-transduction. The flow cytometry samples were fixed with 4% PFA-PBS and analysed with Beckman Coulter’s CytoFLEX S to measure EGFP expression. The proportion of cells expressing EGFP on day 2 post-transduction was compared to the later time points to evaluate the persistence of transgene expression. Cell pellets for site-specific PCR and ddPCR analysis were collected on day 9 post-transduction.

### Antibiotic selection test of transgene expression from T lymphocytes

Primary human T lymphocytes were transduced at a concentration of 7.5K VP/cell based on the vector p24 titre, with three replicate wells of 2x10^5^ cells for each LV. On day 1 post-transduction, the transduced wells were divided in two: the “antibiotic selection” well and the “no selection” well. For antibiotic selection, the T cell medium was supplemented with 300 μg/ml Zeocin (Cat# ant-zn-05, Invivogen, Toulouse, France) starting on day 1 post-transduction. The cell number was counted from pooled aliquots of the replicate wells on days 1, 3, 6, and 10 post-transduction using the NC3000 Viability and cell count assay (Chemometec, Allerod, Denmark). The experiment was repeated on the cells of three donors. Cell pellets for ddPCR analysis were collected on day 10 post-transduction.

### PCR and droplet digital PCR analysis of targeted transgene insertion

Genomic DNA was extracted using the DNeasy Blood & Tissue kit (ref. 69506, Qiagen, Hilden, Germany). The presence of targeted transgene insertion events in MRC-5 cells was first confirmed by site-specific PCR with primers targeting WPRE in the transgene construct (ACGCTATGTGGATACGCTGC) and the genomic DNA 59 bp downstream from the end of the homologous sequence in the homology arms of the LVs (CCCGCTTTCACGGTCTGTAT). The PCR was performed with the Phusion Flash polymerase (ref. F548, Thermo Fisher Scientific, Vilnius, Lithuania), with a program of 98°C 1 min, 35 cycles of 98°C 2 s, 65.4°C 10 s, and 72°C 40 s, and a final extension of 72°C 1 min. The PCR products were further confirmed to be correct by sequencing (Macrogen EZ-Seq service, Amsterdam, the Netherlands) of the PCR products purified from 1% agarose gel (NucleoSpin Gel and PCR Clean-up kit, ref. 740609, Macherey-Nagel, Düren, Germany).

Insertion targeting efficiency was analysed in MRC-5 cells and T cells by ddPCR, by quantifying the total number of LV genome copies, targeted insertion events (NHEJ in both orientations as well as HR), production plasmid carryover and episomal vector copies. The insertion targeting efficiency is reported as the percentage of targeted insertion events of all integrated LV genomes (production plasmid carryover and episomal vector copies subtracted from all LV genome copies). Each assay was run in duplicate with the RPP30 reference standard assay (dHsaCP2500350, Bio-Rad). The primers, probes, consumables and PCR programs used in the reactions are listed in S6–S8 Tables. For droplet PCR, genomic DNA was first digested by BsuRI (ref. ER0151, Thermo Fisher Scientific, Vilnius, Lithuania) or Tru1I (ref. ER0982, Thermo Fisher Scientific, Vilnius, Lithuania) depending on the assay.

### Chromosome collection for karyotyping

The chromosomal integrity of T cells from a single donor transduced with D+H, D+H+HA, INwt, INwt+HA, and RH LVs was analysed by karyotyping. Each LV was used to transduce 4x10^6^ cells at a concentration of 7.5K VP/cell as calculated based on the p24 titre. On day 3 post-transduction, the cells were incubated in Colcemid (L0040-010, Biowest, Nuaillé, France) to keep them in metaphase, followed by an incubation in 0.075 M KCl (0509, J.T. Baker, Radnor, Pennsylvania). Cells were then fixed in 3:1 methanol: acetic acid (HPLC-grade methanol, M/4056/17X, Fisher Scientific, Fair Lawn, New Jersey; glacial acetic acid, AnalR Normapur 20104.334, VWR, Leuven, Belgium). The karyotype was analysed from fifty cells from each fixed chromosome collection sample at the Eastern Finland Laboratory Centre, ISLAB, Kuopio, Finland.

### Statistics

Statistical analysis was done with the GraphPad Prism 5 for Windows, version 5.03, GraphPad Software, San Diego, California.

## Results

### Characterisation of IN-fusion protein LVs

**Fig 1:**
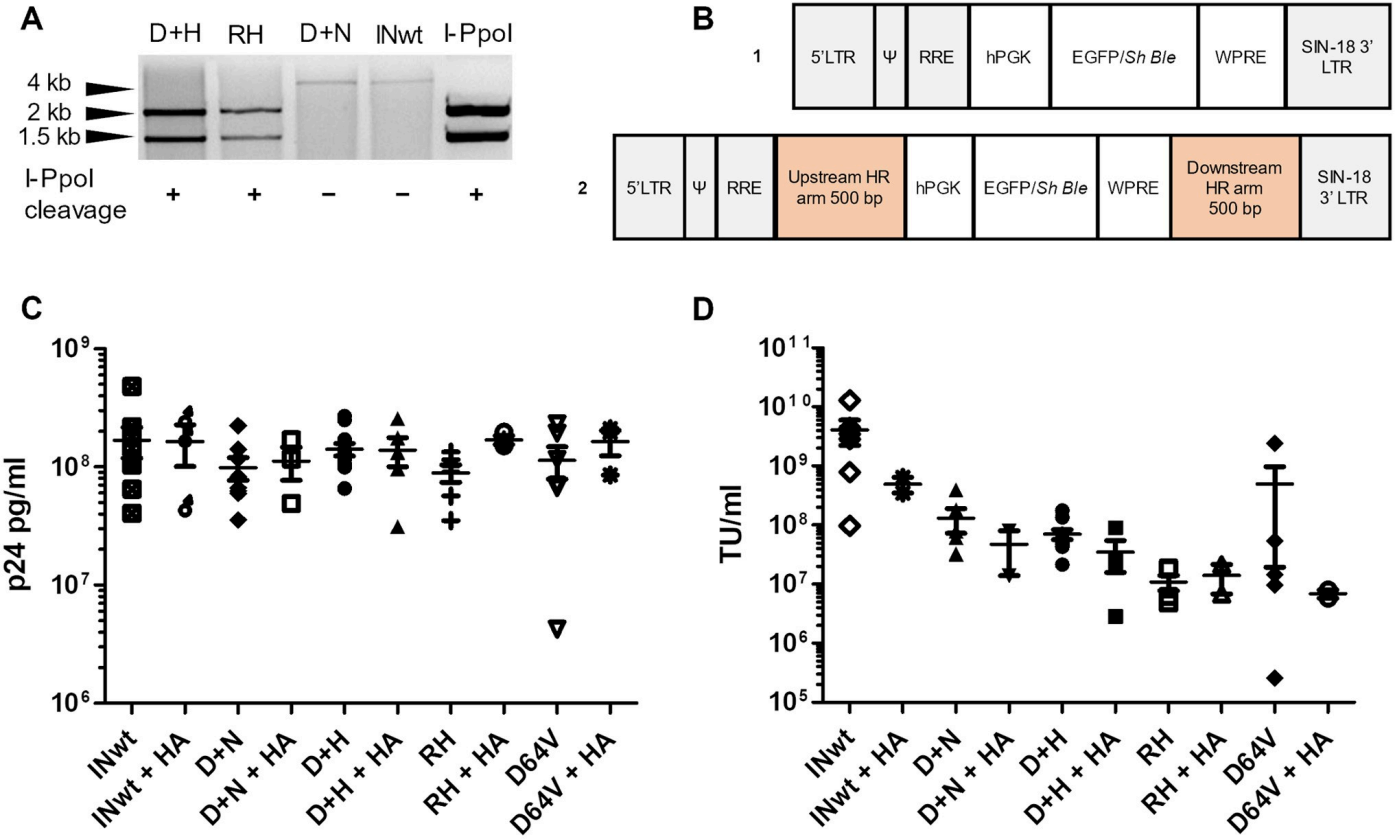
Characterisation of the lentivirus vectors (LVs) used in this study. **A**. IN-I-PpoI fusion protein endonuclease activity of the D+H, RH and D+N LVs *in vitro*. A representative image of cleavage of a plasmid containing an I-PpoI recognition site is shown. INwt and I-PpoI enzyme were included as controls. Expected fragment sizes after Bsu1I linearization and I-PpoI cleavage are 1938 + 1403 bp. **B**. Transgene constructs used in this study: **1**. The standard 3^rd^ generation LV transgene construct; **2**. The 3^rd^ generation LV transgene construct containing rDNA-compatible homology arms (HA LVs). **C**. Particle titres of the LVs used in this study, measured by p24 capsid-protein based ELISA. The mean ± SEM are shown for each LV. **D**. The functional titres of the LVs as measured by flow cytometry from cells transduced with EGFP-LVs. The mean ± SEM are shown for each LV.

The fusion protein LVs used in this study were IN-I-PpoI_R61A+H78A_ (RH), IN_D64V_ + IN-I-PpoI_H78A_ (D+H), and IN_D64V_ + IN-I-PpoI_N119A_ (D+N) (Table 2). We have previously shown that IN-I-PpoI_N119A_ LVs were unable to promote sustained transgene expression and were indistinguishable in this respect from the integration deficient (IN_D64V_) LVs [6]. IN-I-PpoI_H78A_ retained its endonuclease activity but caused a reduction in the viability of transduced cells [8]. The IN_D64V_ *trans*-complementation strategy [14–16] was adopted to rescue the integration proficiency of IN-I-PpoI_N119A_ LVs and to optimize the restriction enzyme activity of the IN-I-PpoI_H78A_ LVs. In this work, the IN-I-PpoI_R61A+H78A_ vectors were produced without IN_D64V_ to ensure maximum concentration of the fusion protein in the vector particles and to enable unequivocal assessment of the enzymatic activities of the fusion protein. The LVs were first characterised for their *in vitro* endonuclease activity and titres. To analyse the endonuclease activity, we performed *in vitro* digestion of a plasmid containing an I-PpoI cleavage site. Crude protein extracts of the D+H and RH LVs cleaved the I-PpoI site, while there was no detectable cleavage of the plasmid with the D+N construct (Fig 1A). This shows that IN-I-PpoI can create the DSBs for targeting integration to its recognition site despite the R61A and H78A mutations.

**Table 2:** Description of the integrase compositions of the LVs used in this study.

Abbreviation	Integrase composition	Function
**INwt**	Wild-type integrase	Unmodified 3^rd^ generation LV control
**D+H**	IN_D64V_ + IN-I-PpoI_H78A_	Integration targeting fusion protein + integration-deficient IN for improved function
**D+N**	IN_D64V_ + IN-I-PpoI_N119A_	
**RH**	IN-I-PpoI_R61A+H78A_	Integration targeting fusion protein
**D64V**	IN_D64V_	Integration-deficient control

Each IN-composition was used to produce two LVs: one carrying a standard transgene construct and one with the HA construct (Fig 1B). The functional and vector particle (VP) based titres were measured for each LV production lot. Neither IN modifications nor the HA transgene construct affected the VP titre (Fig 1C). The functional titre, measured in cells transduced with LVs carrying the EGFP transgene, was lower in the IN-I-PpoI LVs compared to the wild-type IN (INwt) (Fig 1D). LVs generated with the HA construct had lower functional titres in comparison to LVs with the standard transgene construct.

### Stability of the transgene expression by IN-modified LVs depends on the cleavage activity of the IN-fusion protein

**Fig 2:**
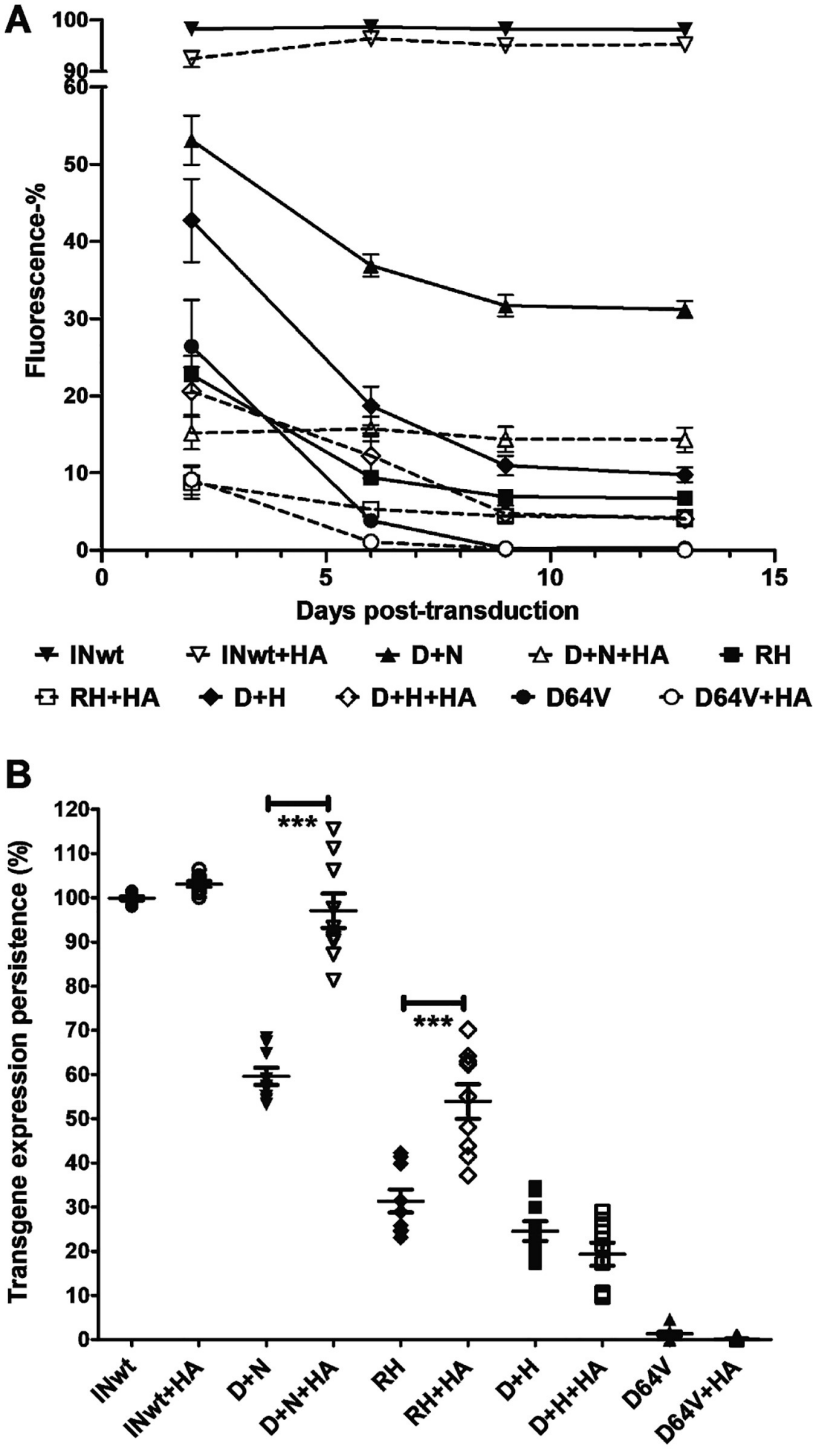
Characterisation of transgene expression stability in IN-I-PpoI transduced MRC-5 cells. **A**. Expression of EGFP-transgene from MRC-5 cells transduced with 7.5K vector particles/cell, measured on days 2, 6, 9 and 13 post-transduction by flow cytometry. Mean ± SEM, n = 9. **B**. Transgene expression persistence on day 13 post-transduction: presented as the percentage of day 2 transgene expression retained at day 13. Mean ± SEM, n = 9. *** = P < 0.001, one-way ANOVA and Tukey’s multiple comparison test.

One of the main benefits of using LVs in gene therapy is their ability to induce long-term expression of the therapeutic transgene. To study the effects of the homology arms and fusion proteins on the transgene expression, we analysed the fluorescence of MRC-5 lung fibroblast cells transduced with LVs carrying the EGFP transgene. All INs were analysed with the standard transgene construct and the HA construct. The cells were transduced with 7.5K VP/cell as measured by their p24 titre, and their EGFP expression levels were followed by flow cytometry measurements from day 2 to day 13 post-transduction. As the transduction was performed based on the vector particle number and not the functional titre, the rate of transgene expression in the beginning of the experiment varied between LVs (Fig 2A). Both the percentage of EGFP expressing cells and the strength of EGFP expression were lower with the HA construct than they were with the regular transgene construct (Fig 2A, S5 Fig). The performance of the LVs was measured by the persistence of transgene expression, which is presented here as the percentage of day 2 transgene expression retained at day 13 (Fig 2B).

All IN-modifications reduced the persistence of transgene expression in comparison to INwt LVs, while the HA transgene construct significantly increased it in the D+N and RH IN-I-PpoI LVs (Fig 2B). The RH+HA had a 53.9±3.7% persistence, significantly higher compared to RH at 31.3±2.5% (P<0.001, Fig 2B). The D+N+HA LV had a 97.0±3.6% persistence on D13, significantly higher compared to D+N at 59.6±1.8% (P<0.001; Fig 2B). Both versions of the D+H retained transgene expression at similar levels. The integration-deficient negative control LV D64V was down to 0% transgene expression on D13 post-transduction, with or without homology arms in the transgene construct, while with INwt LVs, the persistence of transgene expression was nearly absolute. Taken together, high I-PpoI cleavage efficiency in the IN-fusion protein is associated with a lower persistence of transgene expression than what is observed for LVs with a lower or abolished endonuclease activity. Furthermore, while the HA construct increased the persistence of transgene expression in the RH and D+N LVs, the effect was not universal. The observed improvement in expression persistence is therefore dependent on the IN-I-PpoI content of the vector, and likely related to the distribution of the genomic insertion sites of the LVs.

### Targeted transgene insertion into the 28S rRNA I-PpoI site is more efficient in the MRC-5 cell line than in primary CD3+ T lymphocytes

**Fig 3:**
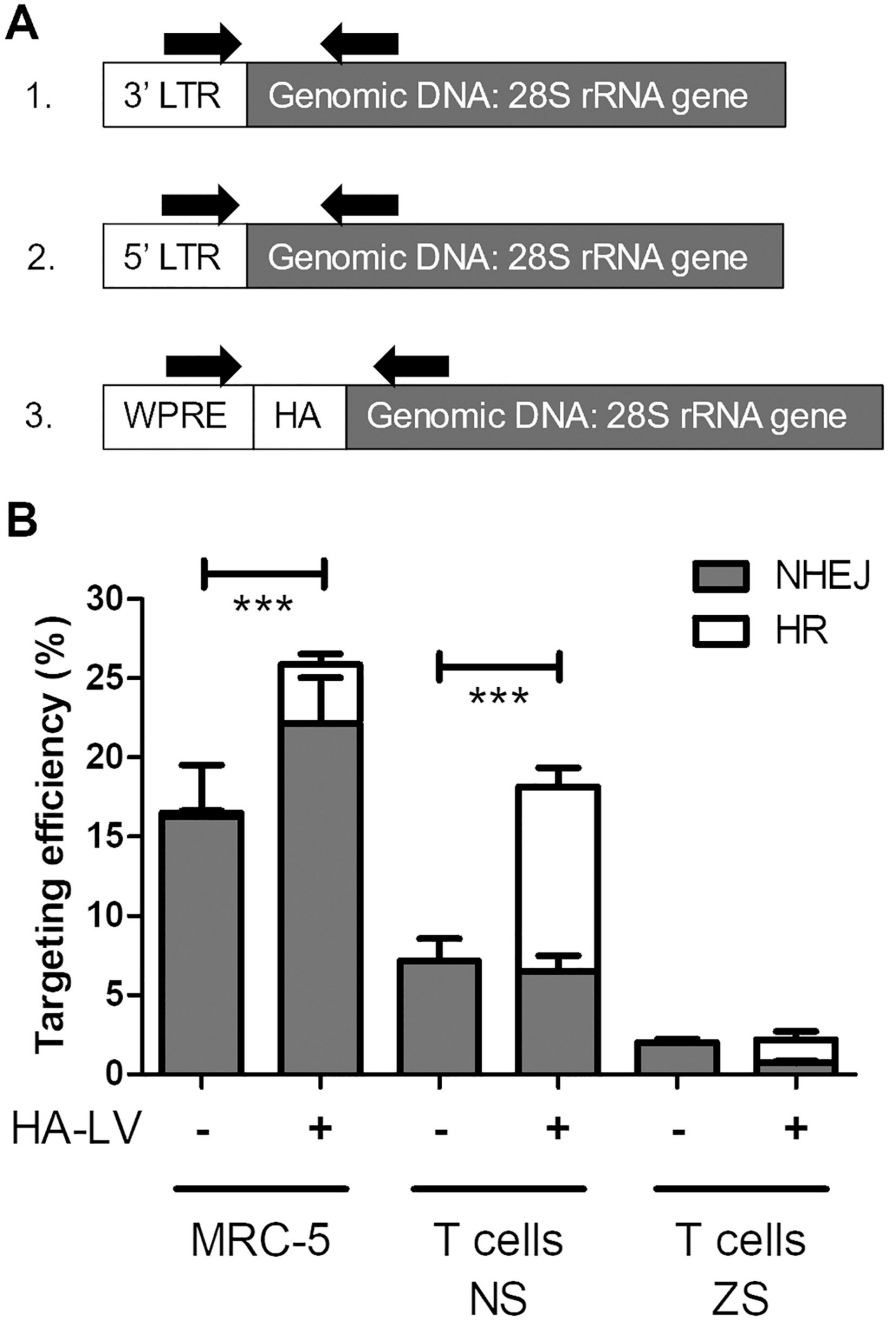
Analysis of targeted insertion by droplet digital PCR (ddPCR). **A**. An illustration of the three separate assays used to measure targeted insertion: 1. Transgene insertion via non-homologous end-joining (NHEJ) in the sense orientation; 2. Transgene insertion via NHEJ in the antisense orientation; and 3. Homologous recombination (HR). Arrows denote primer binding sites. **B**. Overall insertion targeting efficiency of D+H LVs into the 28S rRNA gene as assessed with ddPCR in the MRC-5 cell line (n = 9), primary human T cells with no antibiotic selection (NS) (n = 6) and Zeocin-selected (ZS) T cells (n = 6) on days 9 (MRC-5) and 10 (T cells) post-transduction, measured in samples with standard transgene constructs and with homology arm (HA) transgene constructs. Mean ± SEM. *** = P < 0.001, two-way ANOVA and Bonferroni post-test. See also S1–S3 Tables.

The goal of our fusion protein vectors is to improve the safety of LVs by targeting transgene insertion to the rDNA. To this end, we quantitated the efficiency of targeted insertion into the I-PpoI recognition site in the 28S rRNA gene. As genome editing efficiency can depend on the target cell line, due to factors such as chromatin accessibility, targeted insertion was measured in both the MRC-5 cells and the primary human CD3+ T lymphocytes. Both cell types were transduced with 7.5K VP/cell. For the T lymphocytes, the experiment was repeated on the cells of two donors. For the MRC-5 cells, the experiment was repeated three times. Target site-specific PCR was first used to confirm successful insertion targeting (S1 Fig), then the targeted insertion was measured with ddPCR (Fig 3A).

Of all the LVs tested, only D+H LVs efficiently targeted transgene insertion into the analysed window of 142 bp, and in both analysed cell types, the HA construct increased the insertion targeting efficiency (Fig 3B, S2 Fig, S1–S3 Tables). In the MRC-5 cells, the overall targeting efficiency of the D+H LV without HA was 16.5±3.2% (Fig 3B). The HA construct increased the overall targeting efficiency to 28.2±4.9%. As the majority of the D+H+HA insertion events were via NHEJ, the addition of HA increased the NHEJ-mediated targeting efficiency for this LV. In T lymphocytes, the targeting efficiency of the D+H LV without HA was 7.2±1.3%. Of the D+H+HA LV insertion events, 6.3±0.9% inserted to the target site via NHEJ, and 11.8±0.0% via HR, which increased the overall insertion targeting efficiency to 18.1±1.2%. The targeted insertion efficiency of all other analysed LVs in both cell types was less than 1% (S1 & S2 Tables). These results show that the HA construct alone cannot quantifiably induce targeted transgene insertion to the I-PpoI recognition site in the 28S rRNA gene, but it can enhance insertion targeting when combined with an efficiently cleaving IN-I-PpoI fusion protein. Furthermore, we saw that the cell type affected both the efficiency of targeted insertion, and the type of insertion events detected. In the MRC-5 cell line, overall targeted insertion was more efficient than in the primary T lymphocytes. For the D+H+HA LV in the MRC-5 cells, most of the targeted insertion events were NHEJ integrations, while in the T lymphocytes HR was the dominating pathway for insertion targeting (Fig 3B).

Next, we wanted to assess the efficiency of transgene expression from the target site in the 28S rRNA gene. To this end, insertion targeting was determined in primary human CD3+ T cells transduced with LVs carrying the *sh Ble*-antibiotic resistance gene and subjected to Zeocin-selection from day 1 post-transduction. The cell number was followed throughout the experiment, and in all IN-I-PpoI LVs the cell numbers had surpassed the day 1 numbers by day 10 post-transduction (S3 Fig). In contrast, the integration deficient D64V controls multiplied until day 6 post-transduction, then declined as the episomal transgene expression faded.

A similar insertion targeting efficiency between non-selected and selected cells would suggest strong transgene expression from the target site. However, antibiotic selection greatly reduced the frequency of targeted insertion events in the fusion-protein LVs in comparison to non-selected T cells (Fig 3B, “NS” vs “ZS”). The Zeocin-selected D+H LV samples retained only 28% of the insertion targeting efficiency of the non-selected D+H LVs. With the D+H+HA LV the effect was even more profound, with only 12% of the insertion targeting efficiency remaining after the antibiotic selection. The targeted insertion rates measured for INwt controls remained as they were without the selection (<1%, S3 Table). As the antibiotic selection removes cells lacking efficient transgene expression, the reduced proportion of targeted insertion events in the selected D+H LVs suggests that many transgene copies inserted in the rDNA are not expressed.

### Effective rRNA cleavage induces formation of derivative chromosomes in primary human CD3+ T lymphocytes

Genome editing with the CRISPR/Cas9-system has been shown to induce multiple types of DNA damage, from single-nucleotide variations to large deletions and chromosomal rearrangements [17–20]. Using a nuclease with multiple cleavage sites poses an elevated risk for DNA damage caused by DSB generation [21]. As our fusion-protein LVs target a repeat sequence, we wanted to analyse the effects of the IN-I-PpoI LVs on chromatin integrity. We chose to screen for chromosomal abnormalities by karyotyping metaphase chromosomes, as it is an unbiased genome-wide method for detecting different types of large-scale damage in a single analysis. T lymphocytes from the same donor were used in all analysed samples, and the karyotype was analysed from 50 cells in each sample. The IN-modifications chosen for analysis were RH and D+H, as these I-PpoI modifications showed DNA cleavage activity *in vitro* (Fig 1A). INwt was included as an LV control. The effect of the HA construct was assessed from D+H+HA and INwt+HA transduced samples. A non-transduced (NTD) sample was included as an additional baseline control.

**Fig 4:**
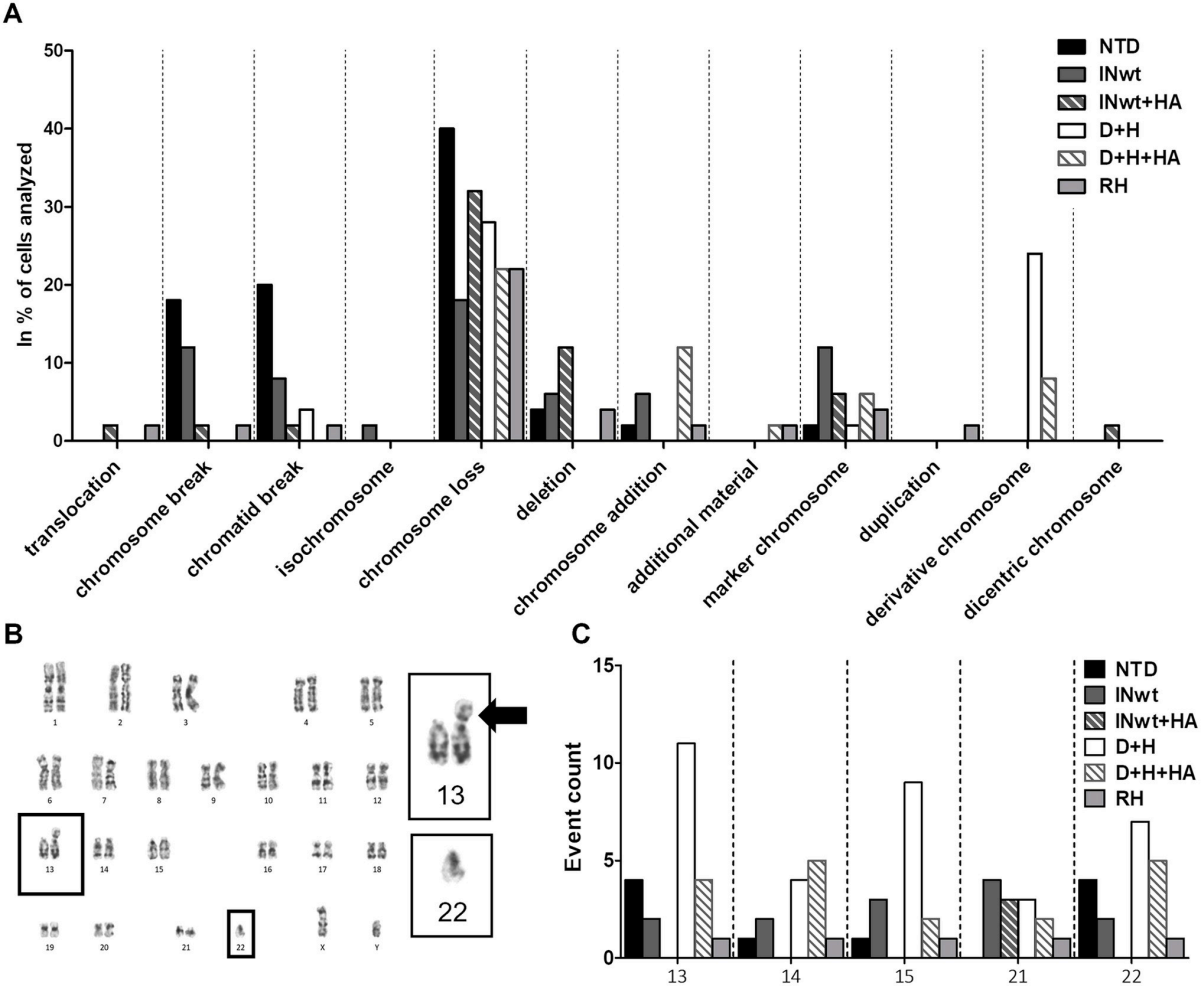
Karyotype changes in T cells as assessed from metaphase chromosomes. **A**. Types of karyotype changes detected in the analysed cells, charted by the percentage of cells where the category’s events were found. See also S4 Table. **B**. An example of the derivative chromosomes found in the D+H karyotyping samples (45, XY, der(13;22)(q10;q10)); the recombined chromosomes 13 and 22, and the derivative chromosome enlarged, with an arrow denoting the derivative chromosome. **C**. Distribution of total structural changes across the chromosomes containing rDNA repeats.

The NTD control showed a high background for chromosome loss, as well as chromosome and chromatid breaks (Fig 4A, S4 Table). The IN-I-PpoI LVs did not increase the number of lost chromosomes or change the pattern of chromosome loss despite the high number of I-PpoI cleavage sites present in multiple chromosomes (S4 & S5 Tables). A translocation event was detected in the RH sample between chromosomes 14 and 17, and in the INwt+HA sample between chromosomes 6 and 9 (Fig 4A, S4 Table). Neither of these translocations was at an I-PpoI recognition site.

Derivative chromosomes, unbalanced translocations between two chromosomes that form a structurally abnormal chromosome [22], were detected in the samples transduced with the IN-I-PpoI_H78A_-containing LVs. They were found in 24% of the analysed cells in the D+H sample, and in 8% of the analysed cells in the D+H+HA sample; this type of event was not found in the other samples (Fig 4A). All derivative chromosomes were Robertsonian translocations (ROBs), rearrangements of the acrocentric chromosomes where a majority of the sequence in the short arms are lost [23]. The short arms of acrocentric chromosomes, which are involved in the formation of ROBs, contain the rDNA repeats where the highest number of genomic I-PpoI cleavage sites are located. This pattern of chromosomal aberrations is therefore clearly linked to the I-PpoI_H78A_ endonuclease contained in the D+H LVs. The frequency of derivative chromosomes in the D+H sample led to the total number of structural changes in the rDNA-containing chromosomes being the highest among the studied vectors (Fig 4C, S4 Table). The lower number of observed derivative chromosomes in the D+H+HA sample was not due to a lower LV dosage, as a vector copy number analysis showed a higher functional vector dose for this LV in comparison to the D+H sample (S4 Fig).

## Discussion

In this study, we have characterised target-site sequence homology containing IN-I-PpoI-fusion protein LVs for their transgene expression and its persistence, their ratio of insertion targeting into the I-PpoI recognition site in the 28S rRNA gene, and their effects on chromosomal integrity. In this study our method of choice for quantifying accurate insertion targeting was ddPCR because it is a quantitative and simple method to measure on-target insertion events. In sequencing-based approaches, stringent filtering can cut out genuine insertion events as possible artefacts. Sampling constraints also often lead to difficulties in estimating the quantity of integration events [24]. With ddPCR, correct targeted insertion events are not likely to be lost. Here we show that a major additional benefit of the ddPCR method is its suitability to the quantitation of HR events, which is not feasible with short-read deep-sequencing methods.

Our previous studies with IN-I-PpoI fusion proteins showed that adding the integration deficient IN_D64V_ into the LV particles with the IN-I-PpoI_H78A_ was essential to dilute the endonuclease activity of the fusion protein-LVs, because alone the IN-I-PpoI_H78A_ caused a cytotoxic amount of DNA cleavage [8]. For the IN-I-PpoI_N119A_-carrying LVs, *trans-*complementation with the IN_D64V_ is required to enable persistent transgene expression [6]. Since the D+N LVs are not observed to cleave I-PpoI sites, we believe they are inserted through NHEJ into DSBs that are naturally present in the parts of the genome that are bound by the I-PpoI_N119A_ part of the IN-fusion protein [7]. The resulting insertion site distribution is safer in comparison to non-modified LVs, which has been our motivation to further develop and study these vectors. Here the new IN-I-PpoI_R61A+H78A_-vectors were produced without IN_D64V_ to ensure maximum concentration of the fusion protein in the vector particles. This facilitates studying the enzymatic activities of the protein and the detection of its possible impacts on the growth rate of transduced cells. We found that the RH LVs do not reduce the growth rate of the transduced cells, and that they induce persistent transgene expression. This suggests that the new IN-I-PpoI_R61A+H78A_ is the most optimal fusion protein we have developed, as it does not require the IN_D64V_ *trans*-complementation to function.

Stable, long-term transgene expression is the hallmark of LVs, and one of the main reasons they are used in gene therapy. For this reason, we assessed the functionality of our LVs by quantifying the persistence of transgene expression. All IN-I-PpoI LVs had a reduced transgene expression persistence compared to INwt LVs, which was expected, as the inclusion of the fusion protein affects the function of the viral IN. The addition of the HA construct significantly improved the persistence of transgene expression in D+N and RH LVs. In fact, the transgene expression persistence for D+N+HA was 97%, which was nearly absolute. This suggests that the HA construct improves the functionality of IN-I-PpoI LVs when used in combination with optimal fusion proteins. However, the HA construct did not make a difference in the integration deficient control, which indicates that the target site homology alone cannot efficiently induce persistent transgene expression. In the D+H LVs, the HA construct also did not affect the persistence of transgene expression. While we have not yet looked into the mechanism of why the homology arms increase transgene expression persistence in D+H and RH LVs, it could be due to increased contacts of the transgene sequence along the genome. The ribosomal DNA repeats have been shown to extensively interact with the rest of the genomic DNA [25], and it is possible that the rDNA sequence present in the homology arms enhances similar interactions of the transgene construct. This could then enable increased integration of the D+N+HA and RH+HA LVs at the genomic sites that they bind.

Transgene insertion targeting efficiency in both the MRC-5 cell line and primary human T cells was clearly highest with the D+H LVs, and the addition of HA to the transgene construct further increased the targeting efficiency of this LV in both cell types. In T cells, the increase was through HR, which was detectable at a much higher rate than in the MRC-5 cells. This is in line with the previous reports that while NHEJ is usually the default pathway of DNA repair in mammalian cells [26, 27], exponentially dividing T lymphocytes may prefer HR [28]. In the MRC-5 cells the HA construct also increased targeted integration through the NHEJ DNA repair pathway. However, the HA construct in other LVs did not target LV integration into the I-PpoI recognition site in the 28S rRNA gene, which suggests that the effect was dependent on the nuclease activity of the IN-fusion protein. The ddPCR assay cannot differentiate between the canonical NHEJ pathway and alternative end-joining, so it is possible that the HA construct increases the use of alternate DSB repair mechanisms [29]. This is further supported by the fact that we only saw this effect in the MRC-5 cell line and not in primary T lymphocytes, as the DSB repair pathway choice has been shown to differ between cell types [30]. Targeted insertion was also analysed from T lymphocytes in which expression of the transgene was forced with an antibiotic selection. This analysis suggested that many transgene copies inserted in the I-PpoI recognition site in the 28S rRNA gene do not become efficiently expressed. The mechanism of the RH LVs’ insertion into the genome is likely similar to that of the D+N LVs. However, the IN-I-PpoI_R61A+H78A_ fusion protein likely cleaves genomic DNA outside of the target site in the 28S rRNA gene more efficiently than the IN-I-PpoI_N119A_, which would explain the superior transgene expression persistence of the RH LVs in the absence of *trans*-complementation.

In the analysis of chromosomal effects of DSB generation, we used primary cells to ensure that the results would be as clinically relevant as possible. Immortalized cell lines often have irregularities in chromosome number and structure [31–33] and the factors used in cell line immortalization affect the control of cell cycle checkpoints and DNA repair mechanisms [34, 35]. In the D+H fusion protein LV samples we found a high number of derivative chromosomes, classified as Robertsonian translocations. With an incidence of 1:1000 [23] such translocations are the most common chromosomal abnormalities found in humans. Although they lead to the loss of the short arms of the acrocentric chromosomes involved, the phenomenon is not considered to have phenotypic consequences [22]. Indeed, the number of rRNA genes can vary greatly both between and within individuals [11] and their inherent surplus seems to counteract potential losses of gene copies due to the natural instability of the rDNA.

The karyotyping analysis showed that RH LVs, although they cleaved the I-PpoI site *in vitro*, caused no detectable karyotype changes attributable to I-PpoI activity. The RH LVs also did not reproduce the high insertion targeting efficiencies of the D+H LVs. Instead, the insertion targeting efficiency and transgene expression persistence of the RH LVs closely resembled the catalytically inactive D+N LVs. These results suggest that the I-PpoI endonuclease activity of the RH LVs was reduced in the cellular environment. The ddPCR method only quantitates targeted insertion into the immediate vicinity of the I-PpoI recognition site in the 28S rRNA gene, but we have previously shown that the D+N LVs target transgene insertion to the entire rDNA and improve the safety of the insertion site profile in many ways in comparison to non-targeted LVs [7]. In the future, we aim to sequence and fully characterise the insertion sites of the RH LVs to see the effects this fusion protein has on the overall safety of the transgene insertion site profile, and to get a detailed insight into the mechanism of improved transgene insertion and expression of our IN-modified LVs when the HA construct is present.

Taken together, the D+H LVs are the most efficient of our targeting LVs in generating DSBs in transduced cells, which enables more efficient transgene insertion targeting to the I-PpoI cleavage site but also manifests as reduced chromosomal integrity. For all genome editing and targeted integration approaches that rely on DSB formation, such as the CRISPR/Cas9-technique, care needs to be taken in analysing the effects of the targeting tool in as comprehensive and unbiased a manner as possible, preferably including the characterisation of the karyotype of the edited cells. While karyotyping cannot be scaled up for a high-throughput analysis or used to detect small-scale mutations, the pattern of chromosomal damage detected in this analysis would have been impossible to detect with other genome editing off-target analysis methods that are currently in use.

## Conclusions

We found that homology arm-containing transgene constructs improved the persistence of transgene expression in LVs that carry IN-I-PpoI fusion proteins with optimally reduced endonuclease activity. The construct also improves targeted insertion into the I-PpoI recognition site in the 28S rRNA gene when used together with an IN-I-PpoI with a high endonuclease activity. However, target site sequence homology alone does not lead to persistent transgene expression or quantifiable targeted insertion of LVs. We also show that ddPCR can be used to quantitate HR of the transgene after LV-mediated gene transfer. The karyotyping approach can be used to assess large-scale chromosomal effects of DSB generation in a genome-wide, unbiased manner in primary cells, and DSB-mediated transgene targeting can lead to drastic impacts on chromosomal integrity. Altogether, our results show that efficient generation of DSBs in the 28S rRNA gene can lead to the formation of derivative chromosomes and that correctly targeted transgenes in this site are not well expressed. We thus conclude that of our IN-I-PpoI LVs, the ones with lower rDNA cleavage activity are preferable to the IN-I-PpoI LVs with high rDNA cleavage activity for use in LV-based gene therapy applications.

## Supporting information

S1 Fig Site-specific PCR for detection of targeted transgene integration. **A**. A representative image of results; lentivirus vectors marked above lanes (NTD = non-transduced control). The samples are flanked by a molecular weight marker (MassRuler DNA Ladder Mix, Thermo Scientific SM0403) and relevant marker bands are annotated on the right. Arrows show expected product sizes from targeted transgene integration; blue arrows show homologous recombination (HR) and non-homologous end-joining (NHEJ) products expected from LVs containing rDNA-compatible homology arms (HA), green arrow shows NHEJ product expected from LVs without HA. **B**. A representative alignment result showing the attachment of the D+H vector’s LTR to the genomic DNA. A product of the site-specific PCR (A) was sequenced and aligned with a template sequence constructed to model the NHEJ-insertion of a vector genome into the I-PpoI cleavage site. The GT-dinucleotide (green annotation) is present at the end of the vector’s 3’ LTR sequence indicative of transgene insertion via NHEJ.
(TIF)

S2 Fig The window of analysis for NHEJ-mediated targeted integration to the I-PpoI recognition site in the 28S ribosomal RNA gene. Integration events outside of this window will not be detected by the ddPCR assay. The genomic DNA binding primer (depicted by the arrow at 236–254 bp) limits the window to 32 bp downstream of I-PpoI cleavage site. Due to sample processing prior to ddPCR, the HaeIII-cleavage site (at 94 bp) limits the window to 110 bp upstream of the I-PpoI cleavage site, leading to a total analysis window of 142 bp. Even in the case of incomplete HaeIII-digestion, the second HaeIII cleavage site (at 15 bp) will limit the window of analysis to a maximum of 221 bp.
(TIFF)

S3 Fig Growth rate of LV-transduced primary human CD3+ T lymphocytes with and without antibiotic selection. **A**. Cell number without antibiotic selection, counted from 3 pooled replicate wells on days 1, 3, 6 and 10 post-transduction (n = 3). **B**. Cell number in samples undergoing zeocin-selection (300 μg/ml) started on day 1 post-transduction. Cells were counted on days 1, 3, 6 and 10 post-transduction from 3 pooled replicate wells (n = 3).
(TIFF)

S4 Fig Vector copy number (VCN) measured from karyotyped cell samples. An aliquot of the cells was collected for VCN analysis as the cells were collected for karyotyping, and VCN was measured by droplet digital PCR (ddPCR). Mean and SEM of two replicate ddPCR runs are shown.
(TIFF)

S5 Fig The EGFP expression levels measured from transduced MRC-5 cells by flow cytometry. (A) The median fluorescence intensity (MFI) is shown from gated LV-transduced cells. The same gating strategy was used for all samples. The INwt LV transduced cells show extremely high fluorescence intensity due to superinfection (vector copy numbers up to 135-fold higher than in IN-I-PpoI samples, see S1 Table). (B) The MFI of IN-I-PpoI LV-transduced cells on a different scale of the Y-axis. The data is presented as mean ± SEM, n = 9.
(TIF)

S1 Table Analysis of targeted integration by droplet digital PCR from MRC-5 cells day 9 post-transduction. (XLSX)

S2 Table Analysis of targeted integration by droplet digital PCR from CD3+ T lymphocytes on day 10 post-transduction (no antibiotic selection). (XLSX)

S3 Table Analysis of targeted integration by droplet digital PCR from zeocin-selected CD3+ T lymphocytes on day 10 post-transduction. (XLSX)

S4 Table Karyotypes analysed from metaphase chromosomes of LV-transduced CD3+ T lymphocytes on day 4 post-transduction. (XLSX)

S5 Table I-PpoI recognition sites in the human genome. (XLSX)

S6 Table Droplet digital PCR assays used in the study. (XLSX)

S7 Table Programs used in ddPCR. (XLSX)

S8 Table Consumables used in ddPCR. (XLSX)

S1 Data Data underlying the findings. (XLSX)

S1 Raw images (PDF)
