# Supplementary figures and images for: Characterization of a new IN-I-PpoI fusion protein and a homology-arm containing transgene cassette that improve transgene expression persistence and 28S rRNA gene-targeted insertion of lentiviral vectors
Source: PLoS One. 2023 Jan 20;18(1):e0280894. doi: 10.1371/journal.pone.0280894 (PMC9858087; doi:10.1371/journal.pone.0280894)

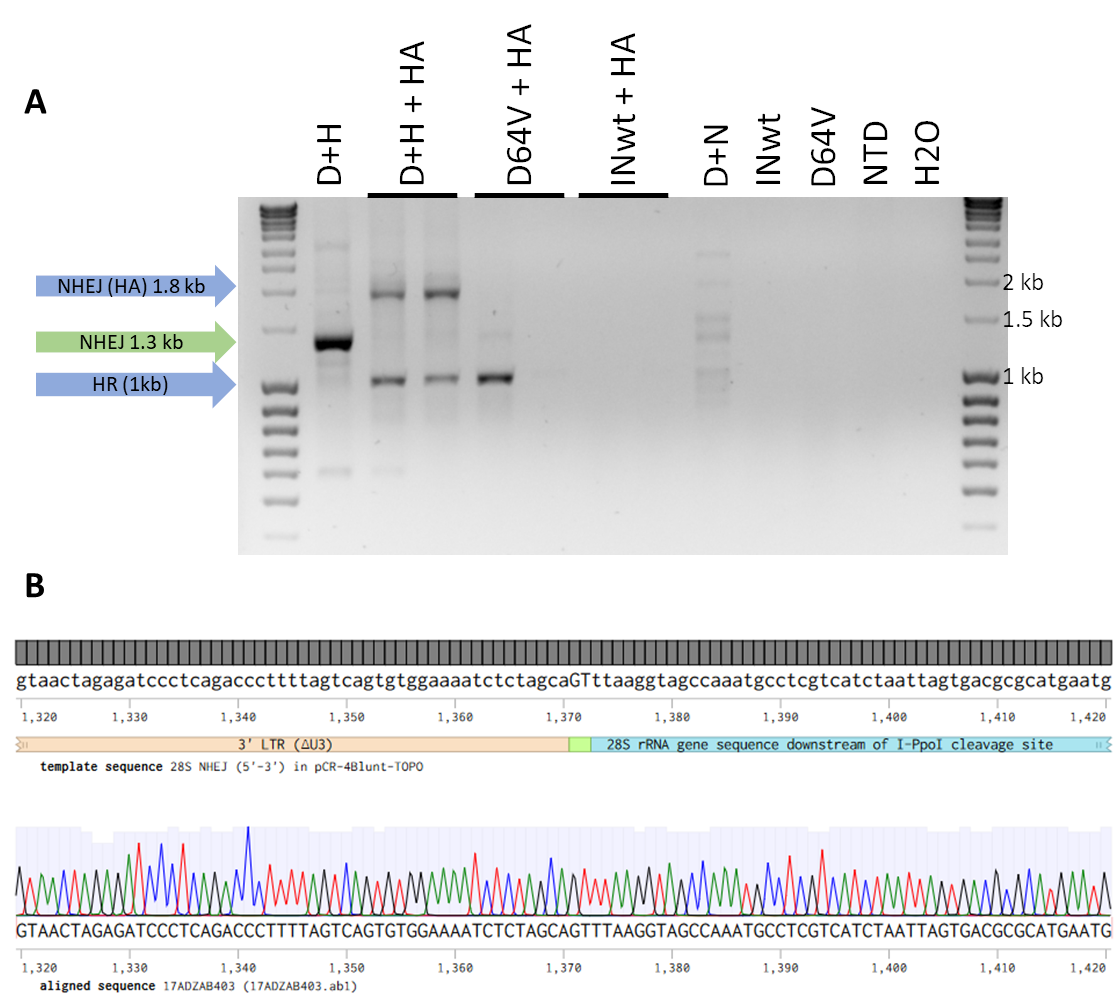

Supplement: S1 Fig — A. A representative image of results; lentivirus vectors marked above lanes (NTD = non-transduced control). The samples are flanked by a molecular weight marker (MassRuler DNA Ladder Mix, Thermo Scientific SM0403) and relevant marker bands are annotated on the right. Arrows show expected product sizes from targeted transgene integration; blue arrows show homologous recombination (HR) and non-homologous end-joining (NHEJ) products expected from LVs containing rDNA-compatible homology arms (HA), green arrow shows NHEJ product expected from LVs without HA. B. A representative alignment result showing the attachment of the D+H vector’s LTR to the genomic DNA. A product of the site-specific PCR (A) was sequenced and aligned with a template sequence constructed to model the NHEJ-insertion of a vector genome into the I-PpoI cleavage site. The GT-dinucleotide (green annotation) is present at the end of the vector’s 3’ LTR sequence indicative of transgene insertion via NHEJ. (TIF) [file pone.0280894.s001.tif]

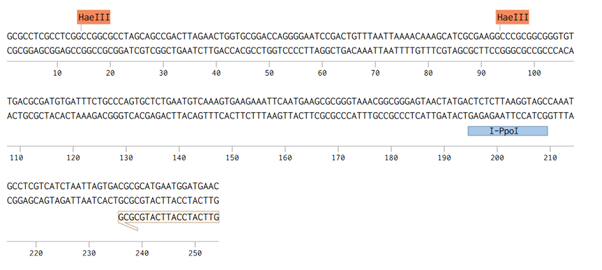

Supplement: S2 Fig — Integration events outside of this window will not be detected by the ddPCR assay. The genomic DNA binding primer (depicted by the arrow at 236–254 bp) limits the window to 32 bp downstream of I-PpoI cleavage site. Due to sample processing prior to ddPCR, the HaeIII-cleavage site (at 94 bp) limits the window to 110 bp upstream of the I-PpoI cleavage site, leading to a total analysis window of 142 bp. Even in the case of incomplete HaeIII-digestion, the second HaeIII cleavage site (at 15 bp) will limit the window of analysis to a maximum of 221 bp. (TIFF) [file pone.0280894.s002.tiff]

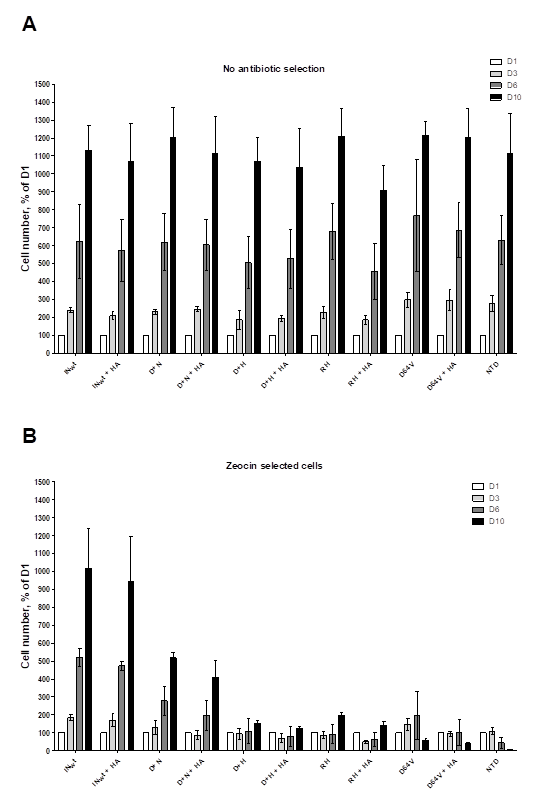

Supplement: S3 Fig — A. Cell number without antibiotic selection, counted from 3 pooled replicate wells on days 1, 3, 6 and 10 post-transduction (n = 3). B. Cell number in samples undergoing zeocin-selection (300 μg/ml) started on day 1 post-transduction. Cells were counted on days 1, 3, 6 and 10 post-transduction from 3 pooled replicate wells (n = 3). (TIFF) [file pone.0280894.s003.tiff]

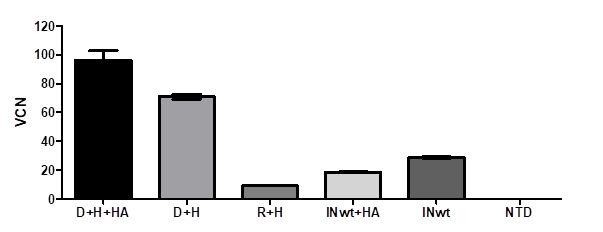

Supplement: S4 Fig — An aliquot of the cells was collected for VCN analysis as the cells were collected for karyotyping, and VCN was measured by droplet digital PCR (ddPCR). Mean and SEM of two replicate ddPCR runs are shown. (TIFF) [file pone.0280894.s004.tiff]

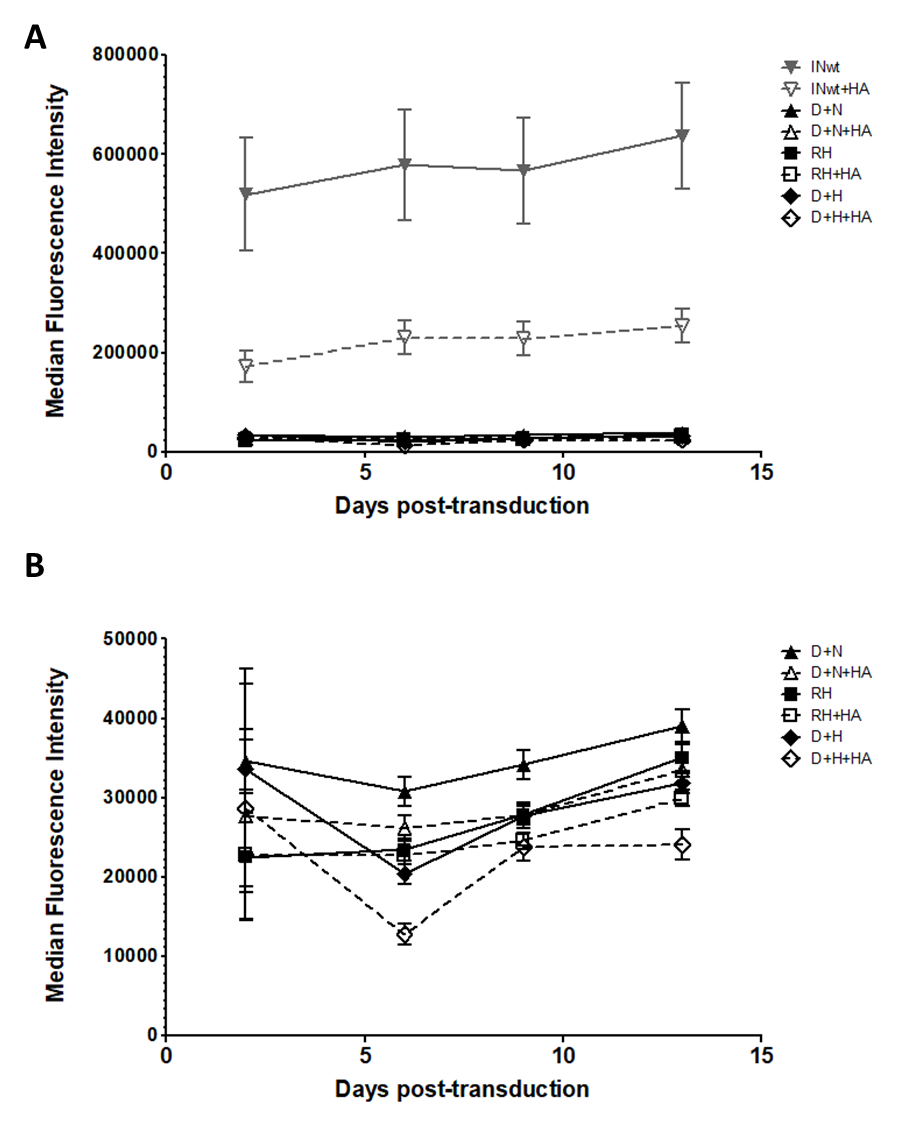

Supplement: S5 Fig — (A) The median fluorescence intensity (MFI) is shown from gated LV-transduced cells. The same gating strategy was used for all samples. The INwt LV transduced cells show extremely high fluorescence intensity due to superinfection (vector copy numbers up to 135-fold higher than in IN-I-PpoI samples, see S1 Table). (B) The MFI of IN-I-PpoI LV-transduced cells on a different scale of the Y-axis. The data is presented as mean ± SEM, n = 9. (TIF) [file pone.0280894.s005.tif]
