# Supplementary material for: Characterization of a new IN-I-PpoI fusion protein and a homology-arm containing transgene cassette that improve transgene expression persistence and 28S rRNA gene-targeted insertion of lentiviral vectors
Source: PLoS One. 2023 Jan 20;18(1):e0280894. doi: 10.1371/journal.pone.0280894 (PMC9858087; doi:10.1371/journal.pone.0280894)
Supplement: S1 Raw images — (PDF) [file pone.0280894.s015.pdf]

1. 2. 3. 4. 5. 6. 7.

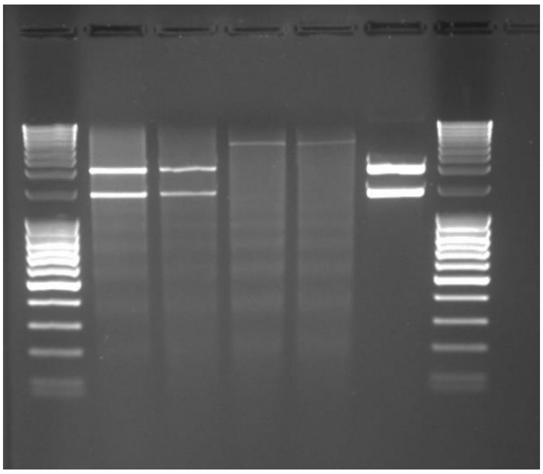

Fig. 1A. 1. MassRuler DNA Ladder Mix (ThermoFisher SM0403), 2. D+H, 3. R+H, 4. D+N, 5. INwt, 6. I-PpoI enzyme control, 7. MassRuler DNA Ladder Mix. 1.5% agarose gel imaged in UV-light with Bio-Rad Gel Doc XR+.

1. 2. 3. 4. 5. 6. 7. 8. 9. 10. 11. 12. 13. 14.

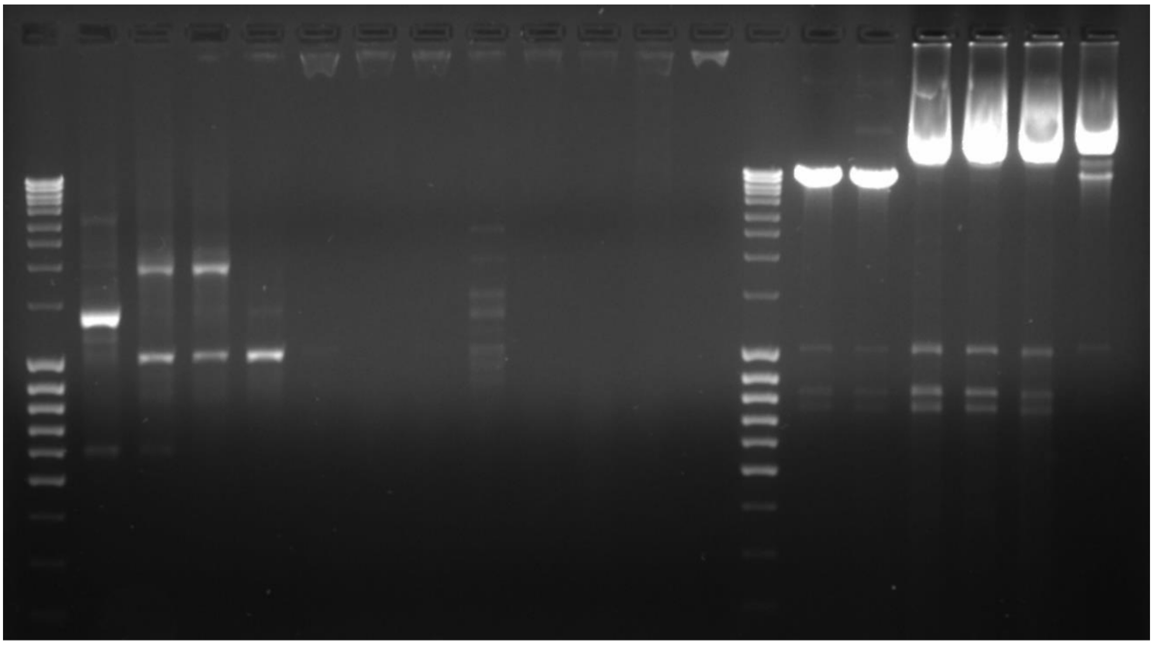

S1 Figure. 1. MassRuler DNA Ladder Mix (ThermoFisher SM0403), 2. D+H, 3. & 4. D+H+HA, 5. & 6. D64V + HA, 7. & 8. INwt+HA, 9. D+N, 10. INwt, 11. D64V, 12. NTD, 13. H2O, 14. MassRuler DNA Ladder Mix. (Lanes beyond from different, unrelated PCR.) 1% agarose gel imaged in UV-light.
